# Contactless Sleep Staging With Radar: A Transfer Learning Approach

**DOI:** 10.1109/OJEMB.2026.3667047

**Published:** 2026-02-23

**Authors:** Daniel Krauss, Robert Richer, Nils Albrecht, Jelena Jukic, Carlos Herrera Krebber, Paul Zwiessele, Alexander German, Alexander Koelpin, Martin Regensburger, Jürgen Winkler, Bjoern M. Eskofier

**Affiliations:** Machine Learning and Data Analytics LabFriedrich-Alexander-Universität 9171 91054 Erlangen Germany; Universitätsklinikum 27168 91054 Erlangen Germany; Institute of High-Frequency TechnologyTechnische Universität 38987 21073 Hamburg Germany; Machine Learning and Data Analytics LabFriedrich-Alexander-Universität 9171 91054 Erlangen Germany; Translational Digital Health GroupInstitute of AI for Health, Helmholtz Zentrum München – German Research Center for Environmental Health 85764 Neuherberg Germany

**Keywords:** Contactless sleep staging, deep learning, heart rate variability, machine learning, radar

## Abstract

Accurate sleep monitoring is essential to assess sleep quality and diagnose sleep disorders. Although sleep laboratories provide precise assessments, they are expensive, labor-intensive, and unsuitable for long-term or large-scale monitoring. Radar-based sensing offers a fully contactless alternative, enabling unobtrusive real-world sleep monitoring. However, the lack of large, labeled datasets has limited the development of robust sleep stage classification models. We address this with transfer learning to improve classification accuracy and generalization to unseen participants within the radar cohort. An LSTM model was pretrained on movement, HRV, and respiratory features from the MESA Sleep dataset ($>$1,100 participants) and fine-tuned using radar data from 44 synchronized polysomnography recordings. Transfer learning increased the Matthews Correlation Coefficient from 0.25 to 0.47 (five-class staging), particularly for Wake, N3, and REM sleep. Future work should explore domain-adaptation across modalities and cohorts. Our results highlight the potential of radar-based sleep analysis for scalable, contactless long-term sleep monitoring.

## I. Introduction

Sleep is an important physiological process that affects a variety of conditions such as physical well-being and cognitive performance [1]. Chronic sleep disruptions are associated with a wide range of health issues, including diabetes or cardiovascular diseases [1], [2]. Moreover, alterations in sleep architecture can serve as an early indicator for neurodegenerative diseases. For that reason, accurate sleep monitoring is crucial to test for sleep disorders and initiate appropriate therapies.

Polysomnography (PSG) is the gold standard for sleep monitoring and is typically conducted overnight in a sleep laboratory [3]. Multiple biosignals, such as electroencephalography (EEG), electrooculography (EOG), electrocardiography (ECG), electromyography (EMG), respiration and oxygen saturation are recorded to assess sleep quality and detect pathological patterns. Sleep stages are scored in in $30-$ or 60-second epochs by trained professionals, primarily based on EEG activity. According to the American Academy of Sleep Medicine (AASM), sleep stages are divided into wake, rapid eye movement (REM), and non-rapid eye movement (NREM). NREM sleep can be further divided into light (N1, N2) and deep sleep (N3) [4]. Despite its precision, PSG is labor-intensive and expensive, making it unsuitable for long-term or large-scale monitoring. Moreover, the unfamiliar, cable-based environment might affect natural sleep patterns, limiting clinical validity of the PSG evaluation [5].

During different stages of sleep, not only brain activity, but also various physiological signals undergo characteristic changes. During NREM sleep, body movement [6], heart rate, blood pressure, and heart rate variability (HRV) [7] decrease. Furthermore, respiration tends to be irregular during wakefulness and REM sleep, but becomes more regular in deep sleep [8], [9].

Physiological changes across sleep stages enable alternative sensing methods beyond EEG-based PSG. Although some consumer devices use wearable EEG [10], most rely on other biosignals (e.g., heart rate, respiration, or movement) from smartwatches or chest straps [11], enabling low-cost, large-scale, home-based monitoring. However, since sleep stages cannot be derived directly from these biosignals, robust machine learning or deep learning classification algorithms are necessary.

Zhai et al. [12] benchmarked machine and deep learning models for unobtrusive sleep staging using actigraphy (ACT) and HRV in mono- and multi-modal settings, finding that the long short-term memory (LSTM) performed best. Other groups used ECG and respiration [13], or combinations of ACT, HRV, and respiration in small cohorts [14], highlighting the high potential of multi-modal sleep stage classification.

Despite these advantages, the practicality of wearables varies depending on the specific sensor system. Although smartwatches are relatively unobtrusive, wearables that rely on ECG electrodes or chest straps can feel intrusive, restricting movement, and potentially interfere with natural sleep patterns. Moreover, wearables require proper placement, consistent usage and regular charging, which can lead to reduced user compliance and variability in signal quality [15].

In contrast, radar-based systems offer a fully contactless and maintenance-free solution for sleep analysis. These systems are based on radio-frequency signals used to capture physiological parameters such as respiration (large chest wall movement), heart sounds (subtle body movements), and gross movements (e.g., repositioning in bed) [16], [17].

As radar signals can penetrate certain materials, the respective sensors can be inconspicuously integrated into the sleep environment [18]. This makes them particularly well-suited for long-term monitoring and populations that may face challenges with wearable technologies, such as elderly individuals or people with movement restrictions.

Kwon et al. [19] reported a $Cohen^{\prime }s\;kappa$ of 0.73 in four-class sleep staging using an attention-based bi-directional LSTM with impulse-radio ultra-wideband radar features. However, the model was trained from scratch without using external datasets, which restricts generalizability to broader populations or real-world conditions. Yang et al. [20] used radar-based nocturnal breathing signals to detect and track Parkinson’s disease with high precision, though their focus was on disease assessment rather than detailed sleep staging.

However, unlike wearables, which have benefited from years of development and large annotated datasets, radar-based sleep monitoring suffers from publicly available labeled data. Although radar can capture relevant physiological signals, they are often subtle and affected by artifacts, making the extraction of relevant information challenging. This, combined with limited availability of annotated radar sleep datasets, challenges the training of deep learning models, which generally rely on large datasets to learn robust representations.

Zhuang et al. [21] developed an end-to-end respiration-based sleep staging framework consisting of a CNN-based feature extractor for FMCW radar range–Doppler representations and a transformer-based temporal predictor. Their model was pretrained on large-scale respiration-belt signals to learn respiratory dynamics and subsequently fine-tuned on raw radar data, achieving a Cohen’s $\kappa$ of 0.62 in four-class staging.

Similarly, Park et al. [22] aligned respiratory spectrograms from wearable belts with UWB radar Doppler maps through domain adaptation and reported a Cohen’s $\kappa$ of 0.64 in four-class staging.

While both approaches improve radar-based sleep staging, they rely on radar-specific spectrograms that require either wideband Doppler information or range–Doppler processing, which are only available in FMCW or UWB radar systems.

Moreover, both studies operate within respiration-focused time–frequency domains and do not address the broader question of whether physiology-level temporal priors learned from large multimodal wearable datasets can improve radar-based sleep staging.

To overcome these limitations, we propose a transfer learning approach that combines movement, HRV, and respiration rate variability (RRV) features from a large sleep dataset (MESA Sleep) for pre-training, followed by fine-tuning on a radar dataset containing 44 overnight PSG recordings. By extracting comparable cardiac, respiratory, and movement features from both datasets, we transfer knowledge learned from sensor-attached biosignals to enhance sleep staging performance with radar data. This aims to improve the accuracy of radar-based sleep staging under limited labeled radar data, and to enhance generalization to unseen participants within the same cohort by importing physiology-level priors from a large wearable dataset. To further contextualize the modality gap between radar-derived and PSG-derived features, we compare the performance of models that use PSG-derived features from the same nights (PSG-only and PSG-transfer learning baselines) with the radar-based classifications.

## II. Materials and Methods

### A. EmpkinS Radar Dataset

The Empatho-Kinaesthetic Sensor Technology (EmpkinS) sleep dataset used in this study was collected during an overnight sleep study conducted at FAU Erlangen-Nürnberg, including 44 participants (Table I) and over 250 hours of sleep data. All participants provided written informed consent under ethics approval 486_20 B (Ethics Committee FAU Erlangen-Nürnberg). Four 61 GHz CW Doppler radar sensors ($fs=1953.125\;\mathrm{Hz}$) [23] recorded data from under the mattress at chest level (Fig. 2). The PSG recordings included a 32-channel EEG, EOG, ECG, EMG (both arms and legs), respiratory belts (abdominal and thoracic), nasal airflow, and measurements of oxygen saturation. Sleep stages were manually scored in $\mathrm{30}\;\mathrm{s}$ epochs by trained clinicians annotated according to AASM-standards [4].

**TABLE I:** Statistics of the *MESA* and the *EmpkinS Dataset*. Age and Total Sleep Duration Per Night (TSD) are Given as Mean $\pm$ SD.

			
**EmpkinS Dataset**			
Dataset	Total (M / F)	Age	TSD (min)
Train	28 (12 / 16)	$37.0 \pm 16.1$	$428.1 \pm 80.8$
Test	08 (02 / 06)	$38.7 \pm 16.7$	$428.6 \pm 78.3$
**MESA Dataset**			
Train	896 (413 / 483)	$68.5 \pm 8.8$	$485.1 \pm 80.3$
Test	224 (93 / 131)	$68.5 \pm 8.4$	$491.6 \pm 76.5$

**Fig. 1.**
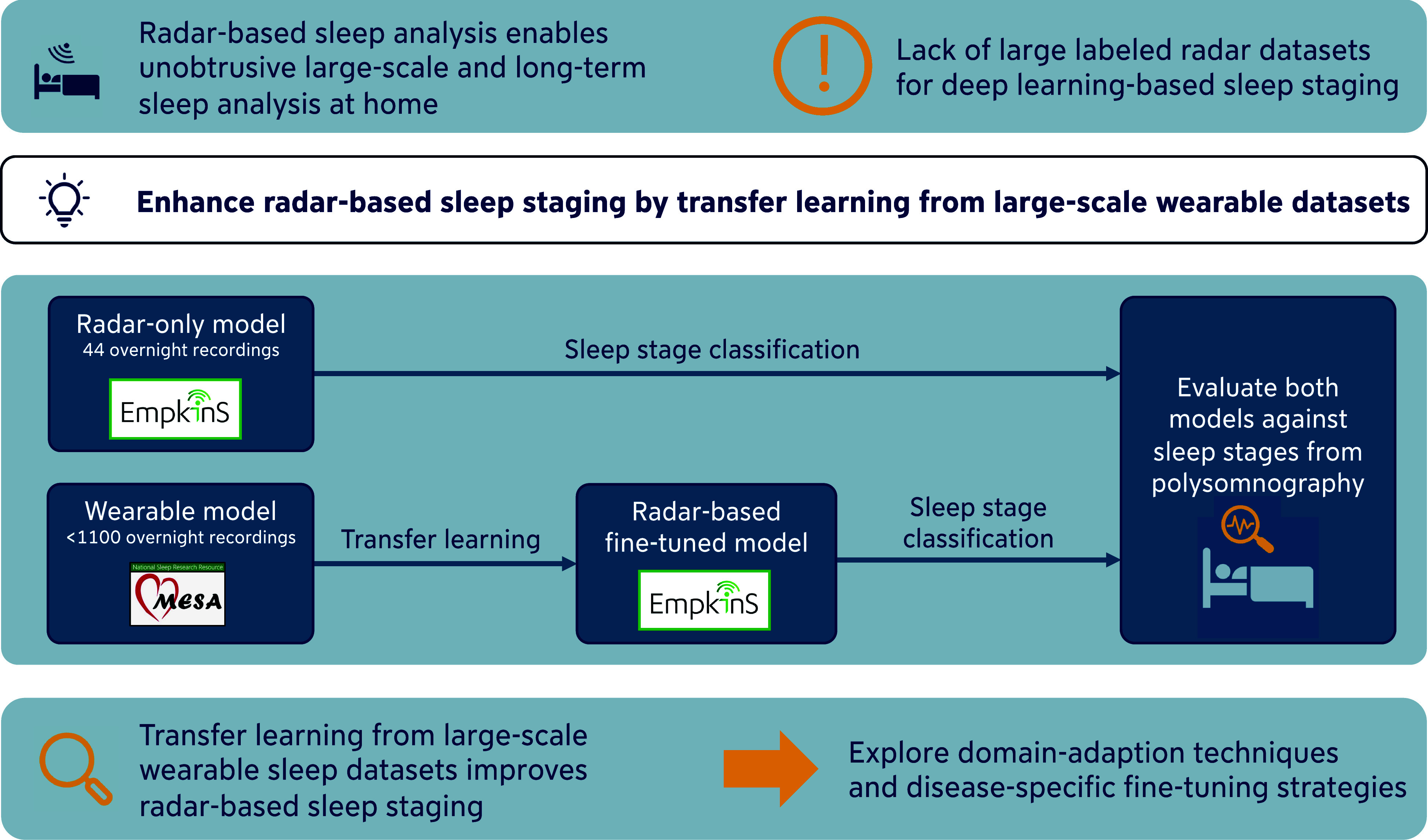
Visual abstract presenting the transfer learning approach and improved radar-based sleep staging performance.

**Fig. 2.**
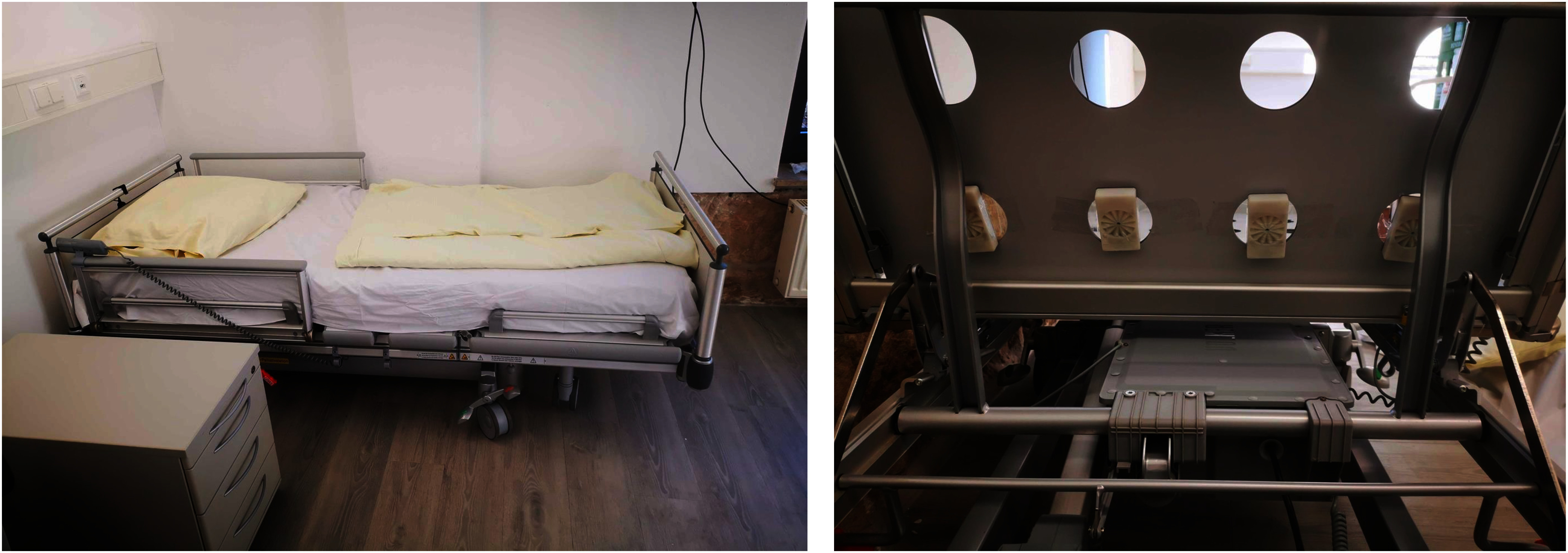
Study setup for radar-based sleep monitoring. (Left) The clinical sleep laboratory environment with a hospital bed used for overnight recordings. (Right) The underside of the bed frame, showing the placement of the radar sensors.

All data streams were synchronized using a simultaneously recorded m-sequence synchronization signal [24]. Eight participants were excluded from the analysis due to poor EEG quality ($n=5$), missing or corrupted radar data ($n=2$), or early-night dropout ($n=1$). Radar fundamentals and sleep stage distribution are provided in the Supplementary Materials I.A-B.

### B. MESA Dataset

The large-scale dataset used in this study is the Multi-Ethnic Study of Atherosclerosis (MESA) Sleep dataset, an open-access dataset that includes PSG and wrist-worn actigraphy (ACT) data from 2,237 participants [25], [26]. Participants underwent unattended PSG recordings that included EEG, EOG, chin EMG, ECG, respiratory effort from thoracic and abdominal inductance plethysmography, leg movements, and pulse oximetry. Sleep stages were scored by trained technicians according to AASM standards [4]. ACT data, collected using wrist-worn devices (Actiwatch Spectrum, Philips Respironics), provided aggregated activity counts in epochs of $\mathrm{30}\;\mathrm{s}$.

We excluded 497 of 2,237 participants due to missing or corrupted PSG or ACT data, missing meta-data, or a total sleep time under 2 hours. Additionally, 620 participants were excluded for low-quality PSG or ACT recordings specified in the meta-data, resulting in 1,120 participants with high-quality overnight sleep data.

## III. Pre-Processing and Feature Extraction

For deep learning-based sleep staging, we extracted large body movement, HRV, and RRV features (Table II). All features were computed in $\mathrm{30}\;\mathrm{s}$ epochs, aligned with the sleep stage labels from the PSG recordings.

**TABLE II:** Overview of Extracted Features and Corresponding Window Sizes Used as Input to the LSTM Model. Features Include Activity Counts, Heart Rate Variability Metrics, and Respiration Rate Variability Metrics. All Features Were Computed in Fixed-Length Sliding Windows as Indicated.

**Feature**	**Description**	**Window Size**
**Actigraphy**		
ACT	Raw activity counts from movement signals	$\mathrm{0.5}\;min$
**Heart Rate Variability**		
MedianNN	Median of NN-intervals (normal-to-normal heartbeats)	$[0.5\;{\&}\;2.5]\;min$
SD2SD1	Ratio between SD2 and SD1 [29]	$\mathrm{0.5}\;min$
VLF	Power in the very low-frequency band (0.003 to 0.04 Hz)	$\mathrm{2.5}\;min$
LF	Power in the low-frequency band (0.04 to 0.15 Hz)	$\mathrm{2.5}\;min$
HF	Power in the high-frequency band (0.15 to 0.4 Hz)	$\mathrm{2.5}\;min$
LF/HF ratio	Ratio of power in low-frequency to high-frequency band	$\mathrm{2.5}\;min$
Total Power	Total power across all frequency bands	$\mathrm{2.5}\;min$
**Respiration Rate Variability**		
MedianBB	Median duration of breath-to-breath (BB) intervals	$\mathrm{2.5}\;min$
LF	Power in the low-frequency band (0.04–0.15 Hz)	$\mathrm{2.5}\;min$
CVBB	Coefficient of variation of BB intervals (standard deviation / mean)	$\mathrm{2.5}\;min$
MCVBB	Mean-centered coefficient of variation of BB intervals (mean absolute deviation / median)	$\mathrm{4.5}\;min$

The features derived from the PSG datastreams of the *MESA* and the *EmpkinS dataset* were extracted as previously described by our group [27], while the same features from the *EmpkinS* radar data were derived as follows:

### A. Large Body Movement Features

Large body movement was extracted from the *in-phase* radar signal of each of the four radar nodes by first applying a $\mathrm{10}\;\mathrm{s}$ moving average filter to reduce noise while preserving movement trends. The signal was then standardized to zero mean and unit variance before computing its first-order derivative to estimate movement intensity. The resulting derivative signal was then normalized to the range $[0, 1]$ within each recording. A movement intensity threshold of 0.2 was applied, setting values below this threshold to 0.0 to suppress minor fluctuations and noise. Finally, the processed signal was segmented into $\mathrm{30}\;\mathrm{s}$ epochs and mean values within each epoch were computed to derive an aggregated movement feature. To obtain a single feature that combines all recorded movements, we computed the sample-wise maximum across radars.

### B. HRV Features

HRV features were extracted by detecting individual heartbeats from the *in-phase* and *quadrature* channels of the raw radar signals using a pre-trained bidirectional LSTM model from previous work [23]. This model outputs a probability value for each time sample, indicating the likelihood of a heartbeat. Since the dataset includes four radar nodes, each positioned slightly differently under the bed relative to the participant, we fused the outputs to improve detection robustness. Specifically, for each time sample, we selected the maximum probability value across the four radar nodes, ensuring that if our algorithm detected a heartbeat at any radar node at a given time point, this detection was retained in the final combined probability signal. Subsequently, heartbeats were identified by applying a peak detection algorithm to this fused probability signal, and the resulting beat-to-beat intervals were used to compute eight different HRV features.

### C. RRV Features

RRV features were extracted by deriving a respiration signal from the *in-phase* radar signal. First, a second-order Butterworth low-pass filter ($\mathrm{0.5}\;\mathrm{Hz}$ cutoff) was applied to remove high-frequency artifacts. To enhance respiration-induced low-frequency modulations, we applied the Hilbert transform, generating a smooth envelope that accentuated the breathing pattern. Individual breaths were then detected using a peak-finding algorithm and the resulting respiration cycles were used to compute four different RRV features [28].

### D. Cross-Modal Feature Correlation Analysis

To examine the feature-level agreement between radar- and PSG-derived signals, we computed participant-wise feature correlations (Supplementary Materials III). Medium-to-strong correlations were observed for timing-based features (median NN-intervals, MedianBB) and movement (Scatterplots in Fig. 5), while spectral HRV indices exhibited weak correlations. The complete correlation matrix is provided in the Supplementary Materials (Figure S6).

**Fig. 3.**
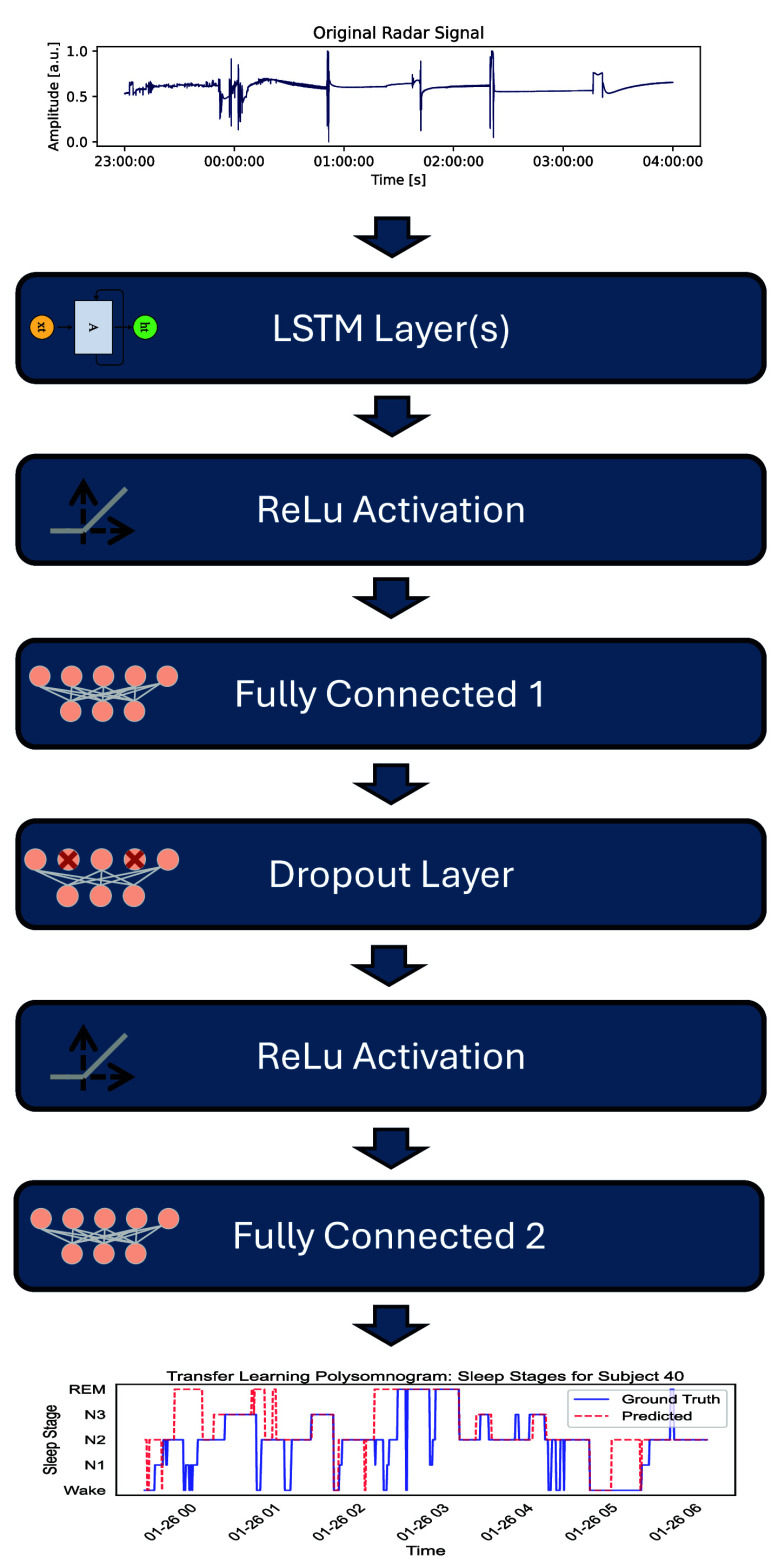
Layer-based sequence classifier to predict sleep stages from physiological features.

**Fig. 4.**
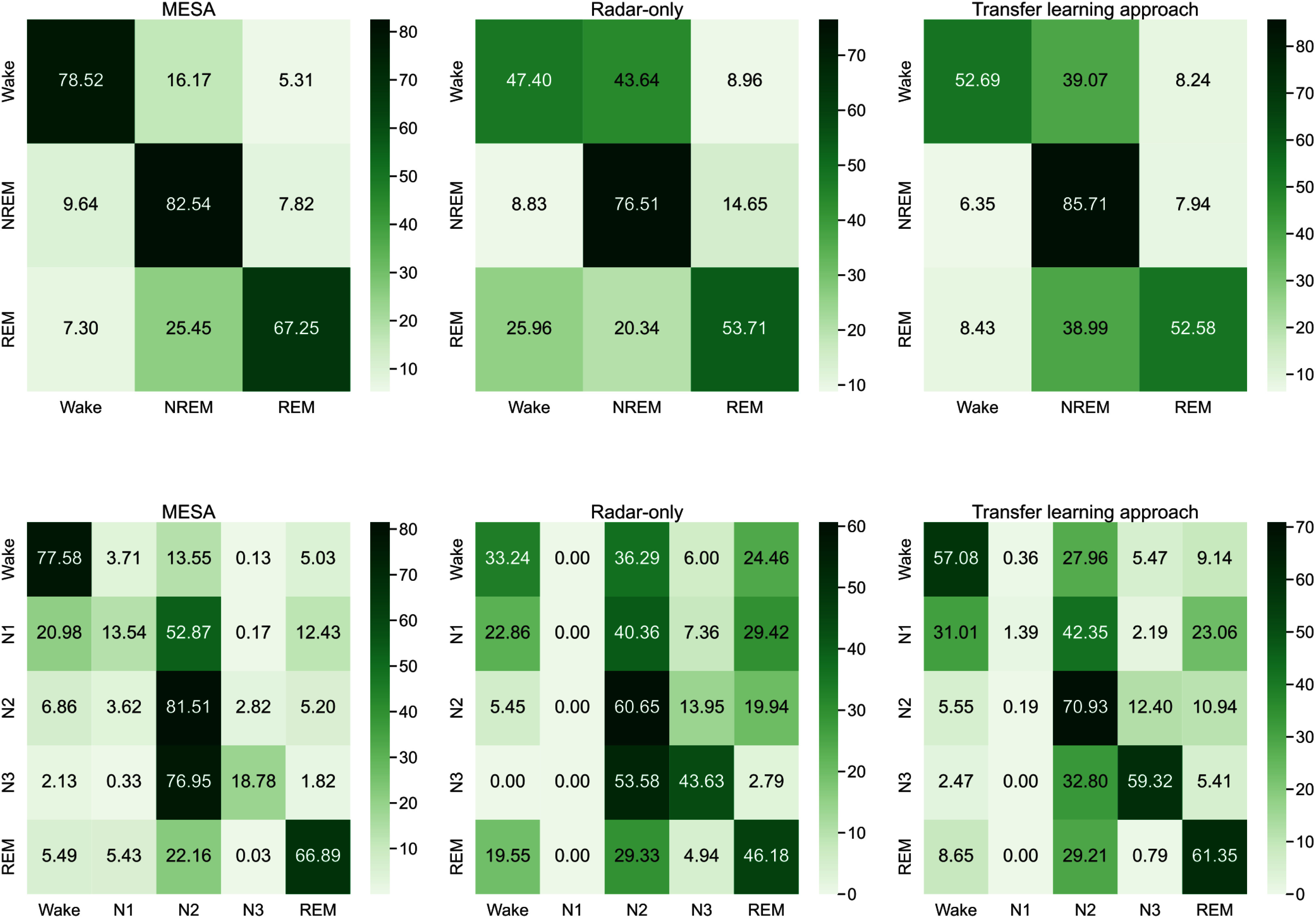
Confusion matrices for sleep stage classification in the three-class scheme (Wake, NREM, REM) and according to AASM standards (Wake, N1, N2, N3, REM) for the radar-only approach (*EmpkinS Dataset*), the transfer learning model (*EmpkinS Dataset*), and the *MESA sleep dataset* as a reference.

**Fig. 5.**
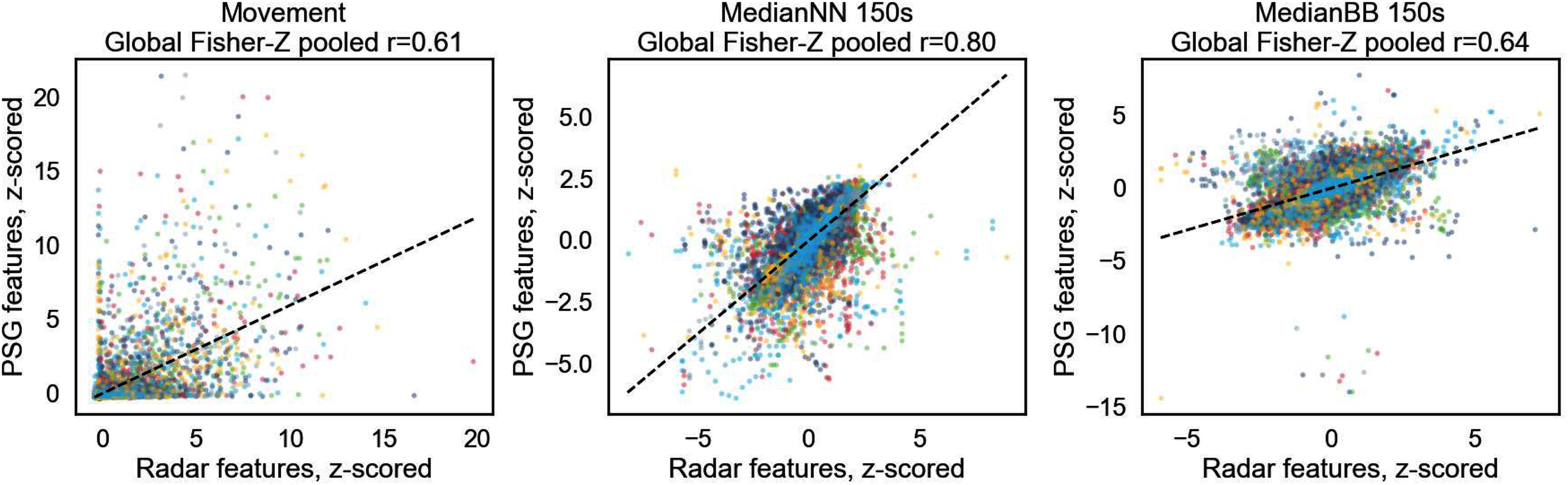
Scatterplots for one respiratory, cardiac and movement feature extracted both from radar data and from sensor-attached data of the *EmpkinS dataset*. Each color indicates one participant.

### E. Model Training

We applied an LSTM-based sequence classifier to map per-epoch features to sleep stages (Fig. 3). Architectural details, equations, optimization, and evaluation procedures are provided in the Supplementary Materials.

We explored two sleep staging schemes in our experiments:

• Five-class staging based on AASM guidelines (Wake, N1, N2, N3, REM) [4].
• Three-class staging (Wake, NREM, REM), where NREM aggregates N1, N2 and N3.

Under the same protocol, we conducted five experiments to assess the performance of our transfer learning approach:

• *MESA reference:* The training was performed exclusively on the *MESA dataset* to learn generalizable sleep patterns from a large-scale sleep dataset before transferring knowledge to radar-based sleep staging.
• *Radar models:* A radar-only baseline was established by training the model after random initialization directly on the radar features *(EmpkinS dataset)*. Additionally, we applied transfer learning by fine-tuning the best-performing MESA-model.
• *EmpkinS PSG models:* We also established a baseline for sleep stage classification using features derived from the concurrent PSG recordings. Similarly to the radar models, we applied transfer learning by fine-tuning the best-performing MESA-model.

### F. Transfer Learning

The LSTM network was initialized with the pre-trained weights from the MESA model, and training continued using features derived from the EmpkinS dataset to adapt the model to dataset/radar-specific features. The fine-tuned model was then evaluated using the held-out test set.

## IV. Results

Table III summarizes the performance for three-stage (Wake, NREM, REM) and five-stage (Wake, N1, N2, N3, REM) classification tasks for the five experiments.

**TABLE III:** Algorithm Performance for Sleep Staging, Reported for Both the Five-Class Scheme Based on AASM Guidelines (Wake, N1, N2, N3, REM) and the Simplified Three-Class Scheme (Wake, NREM, REM). Metrics Were Computed Individually for Each Participant and are Reported as Median Values With Interquartile Ranges (IQR).

	**Accuracy** [%]	**F1-score** [%]	**MCC**	**Precision** [%]	**Recall** [%]	**Specificity** [%]
**Classification scheme 1: Wake / NREM / REM classification**						
**MESA**	$80.95 (11.27)$	$81.16 (11.48)$	$0.67 (0.19)$	$83.01 (08.68)$	$80.95 (11.27)$	$85.26 (12.32)$
**EmpkinS PSG-only**	$62.60 (17.97)$	$55.56 (16.05)$	$0.34 (0.22)$	$55.91 (15.68)$	$62.60 (17.97)$	$74.54 (14.22)$
**EmpkinS Radar-only**	$69.89 (13.25)$	$71.38 (14.09)$	$0.39 (0.22)$	$77.17 (12.48)$	$69.89 (13.25)$	$66.20 (17.84)$
**EmpkinS PSG Transfer learning**	$79.95 (07.25)$	$81.96 (06.08)$	$0.63 (0.08)$	$84.00 (05.88)$	$79.95 (07.25)$	$80.88 (07.22)$
**EmpkinS Radar Transfer learning**	$78.66 (05.30)$	$77.25 (06.32)$	$0.50 (0.07)$	$79.12 (06.43)$	$78.66 (05.30)$	$71.98 (08.39)$
**Classification scheme 2: Classification according to AASM Standards (Wake / N1 / N2 / N3 / REM)**						
**MESA**	$68.04 (12.43)$	$65.16 (13.28)$	$0.54 (0.15)$	$68.65 (12.37)$	$68.04 (12.43)$	$89.01 (06.16)$
**EmpkinS PSG-only**	$51.33 (15.35)$	$50.45 (13.63)$	$0.37 (0.17)$	$58.14 (06.55)$	$51.33 (15.35)$	$77.05 (11.62)$
**EmpkinS Radar-only**	$48.31 (05.60)$	$46.16 (02.94)$	$0.25 (0.08)$	$46.22 (04.62)$	$48.31 (05.60)$	$77.88 (03.52)$
**EmpkinS PSG Transfer Learning**	$67.05 (10.29)$	$66.28 (11.82)$	$0.60 (0.11)$	$72.44 (09.15)$	$67.05 (10.29)$	$88.17 (06.45)$
**EmpkinS Radar Transfer learning**	$61.43 (06.80)$	$58.68 (06.36)$	$0.47 (0.08)$	$62.22 (08.85)$	$61.43 (06.80)$	$83.66 (03.52)$

### A. Classification Scheme 1: 3-Class Staging

In the simplified three-class task, the transfer learning approach outperformed the radar-only baseline across all metrics. The radar-only model achieved an MCC of $0.39\;(0.08)\;(median\;interquartile\;range\;(IQR))$, while transfer learning improved this to $0.50\;(IQR: 0.07)$.

To directly compare the radar-based approach with the sensor-attached one, we evaluated two additional baselines on the *EmpkinS dataset:* PSG-only achieved an MCC of $0.34\;(0.22)\;(median\;(IQR))$. With transfer learning, performance increased to an MCC of $0.63\;(0.08)\;(median\;(IQR))$.

The MESA model achieved the highest overall performance with an MCC of $0.67\;(0.19)\;(median\;(IQR))$. However, this primarily serves as an upper benchmark, given the larger dataset size and different sensing modality.

The confusion matrices (Fig. 4) show improvements across most stages. Wake and NREM detection increased from $47.40\;\%$ (radar-only) to $52.69\;\%$ (transfer learning) and from $76.51\;\%$ (radar-only) to $85.71\;\%$ (transfer learning), respectively, while the detection of REM sleep remained comparable (transfer learning: $52.58\;\%$, radar-only: $(53.71\;\%)$).

### B. Classification Scheme 2: 5-Class Staging

Among the same participants, the radar-only model achieved an MCC of $0.25\;(0.08)$ (median (IQR)), while the transfer learning approach largely improved performance to an MCC of $0.47\;(0.07)\;(median\;(IQR))$.

Using PSG-derived features from the *EmpkinS dataset*, the PSG-only model reached an MCC of $0.37\;(0.17)\;(median\;(IQR))$. With transfer learning, the approach improved to of $0.60\;(IQR: 0.11$), exceeding both the radar-transfer learning model ($MCC = 0.47$) and the MESA reference ($MCC = 0.54$).

For reference, the model trained exclusively on the large-scale *MESA dataset* achieved the highest performance, with an MCC of $0.54\;(0.15)\;(median\;(IQR))$.

Transfer learning increased detection rates across all sleep stages. Specifically, Wake detection improved from $33.24\;\%$ to $57.08\;\%$, N2 from $60.65\;\%$ to $70.93\;\%$, REM from $46.18\;\%$ to $61.35\;\%$, and N3 sleep from $43.63\;\%$ to $59.32\;\%$. The N1 stage remained challenging across all models, with few correctly classified epochs.

## V. Discussion

A key finding is the consistent improvement across all evaluation metrics when fine-tuning the MESA model on radar data, particularly in distinguishing individual sleep stages.

Large, public datasets that combine PSG and radar are scarce, so radar-only models are prone to overfitting and class imbalance. In contrast, large wearable datasets are publicly available, and capture the same latent physiology through different sensing mechanisms. These sensing differences introduce a domain mismatch: radar measures cardio-mechanical micromotions and is more susceptible to mattress attenuation and body-movement artifacts, whereas, for example, ECG measures electrophysiological excitation.

Pretraining on a large, heterogeneous wearable dataset exposes the model to a wide range of sleep architectures, body types, and behaviors, enabling it to learn stage-specific temporal priors, inter-subject invariances, and robust feature connections between movement and HRV/RRV.

When transferred to radar, these generic representations regularize the model, lowering the effective sample complexity and counteracting class imbalance in our smaller radar cohort. Subsequent fine-tuning calibrates the decision boundaries to radar’s modality-specific characteristics, mattress attenuation, and motion artifacts, thus closing a part of the domain gap. In our data, this results in consistent gains in all metrics, with the largest improvements for Wake, N3, and REM.

To contextualize the remaining modality gap, we trained models using PSG-derived features from the *EmpkinS dataset* for the same participants. These models, especially with transfer learning, outperformed their radar counterparts (Table III). Across both classification schemes, the difference between PSG-transfer learning and radar-transfer learning remains similar when measured by MCC (0.13 in both the 3-class and 5-class settings), while accuracy and F1-score show slightly larger discrepancies in the 5-class case. This is expected as finer distinctions between N1, N2, and N3 rely more on subtle subtle changes in autonomic regulation and sleep architecture that might be better represented given the higher signal fidelity of ECG-based HRV and belt-based respiration and the reduced susceptibility to movement-related artifacts

Accordingly, we treat radar-derived HRV as a correlate of autonomic activity rather than a substitute for ECG-HRV. However, robust dorsal placement, which matches our under-mattress setup, has shown comparatively high resilience for radar-cardiac sensing [30]. This domain mismatch leads to classification errors when the radar features are directly applied to the pre-trained model.

The correlation analysis comparing radar- vs. PSG-derived features supports this finding, as timing-based cardiac and respiratory features (median-NN & breath-to-breath intervals) transfer best across modalities ($r=0.80 - 0.64$, Fig. 5), while spectral HRV showed weak cross-modal correlations ($r=0.01 - 0.20$, Supplementary Materials, Figure S7). Existing transfer learning approaches using radar data differ fundamentally from ours. Zhuang et al. and Park et al. achieved Cohen’s $\kappa$ values of 0.62 and 0.64, respectively, in four-class sleep staging—performance comparable to our PSG transfer learning results (MCC = 0.60 in three-class and 0.63 in five-class staging). However, these studies rely on large FMCW or UWB radar datasets with high-resolution range–Doppler or Doppler spectrograms and end-to-end architectures optimized for such rich inputs.

In contrast, our smaller dataset ($n=44$) is based on under-mattress CW radar, which cannot provide the spectrogram inputs required for end-to-end models. Although CW radar yields raw respiration and micromotion signals, their morphology differs substantially from ECG and respiratory belt waveforms due to mattress attenuation and superimposed body movements, creating a domain mismatch that limits cross-modal waveform learning. We therefore used a different approach by transferring physiology-level features instead of radar-specific representations.

The N1 stage remains the most challenging to classify, even for the MESA model. One reason is the low prevalence of N1 sleep in the dataset ($7.3\;\%$, approximately $\mathrm{250}\;min$), which limits the available training data. More importantly, N1 is a transitional stage that lacks distinct physiological patterns that clearly separate it from neighboring stages such as Wake and N2. Because of these challenges, several prior works report four-class staging by merging N1 with N2 [21], [22]. Future work might focus on improving N1 detection by incorporating longer temporal context or by refining radar-derived HRV and RRV estimates to better capture the subtle autonomic changes associated with this stage. However, previous studies have reported the lowest inter-rater reliability for N1 in manual sleep staging ($Cohen^{\prime }s\;kappa \approx 0.24$) [31], resulting in an inherently challenging training environment for automated approaches.

Interestingly, N3 sleep detection improved in both radar-only and transfer learning models compared to the MESA base model. Potentially, N3 sleep, which is characterized by minimal movement and stable autonomic regulation (e.g., slow heart rate, regular breathing) [6], [7], [9], is captured more effectively through radar-derived full-body motion signals. Radar sensors can detect a wide range of movements (e.g., thoracic and abdominal excursions, limb repositioning, postural adjustments), potentially making them more sensitive to the transition into and maintenance of deep sleep states. In contrast, the *MESA dataset* relies on wrist-worn actigraphy, which captures only localized motion at a single extremity and may be less sensitive to reduction in whole-body movement during N3 sleep. Although we cannot conclusively attribute these improvements to differences in movement assessment, the combination of physiological generalization learned from the large *MESA dataset* and the adaptation to radar-specific movement dynamics through fine-tuning might have contributed to this performance boost. Future studies should assess model performance in more detail with explainable AI techniques such as Deep Shap [32], saliency maps [33], or feature ablation studies.

REM sleep is characterized by muscle atonia (little to no movement) but high HRV fluctuations [7], [34]. The MESA base model effectively captured the REM-specific HRV dynamics, which, when fine-tuned with radar data, improved its recognition. However, misclassification between REM and NREM persisted, suggesting that radar-based HRV estimation remains noisier and less precise compared to ECG-derived measures.

## VI. Conclusion and Outlook

This study demonstrated that transfer learning from models trained with sensor-attached bisoginals improves radar-based sleep staging compared to training on radar data alone.

However, the performance remains below that of the MESA base model, likely to the smaller cohort. Furthermore, residual domain mismatch between ECG and radar-derived features (and between wrist actigraphy and radar movement) restricts full transferability. Future work should therefore explore domain adaptation techniques to better align feature spaces across sensing modalities to further enhance generalizability. Future work should also assess external cross-dataset generalizability to independent radar cohorts, which would require multi-site radar–PSG datasets.

Furthermore, larger under-mattress datasets might enable the development of end-to-end models on raw radar-derived biosignals. However, the morphology of raw CW radar heart sound, respiration, and movement signals differs substantially from from actigraphy, ECG, or respiration belt waveforms making it challenging to close the domain gap.

Although this study focused on healthy participants, pathological conditions such as REM sleep behavior disorder can alter the typical physiological characteristics of REM sleep, increasing the risk of misclassifications especially in models trained predominantly on data from healthy individuals [35]. Future research should therefore explore disease-specific fine-tuning strategies to adapt sleep staging models for clinical applications.

## Code Availability

In order to ensure transparency and reproducibility of our results, we made the code used in this study available on GitHub at https://github.com/mad-lab-fau/sleep_analysis.

## Author Contributions

DK: Conceptualization, Data curation, Formal Analysis, Investigation, Methodology, Project administration, Software, Validation, Visualization, Writing – original draft, Writing – review & editing; RR: Conceptualization, Supervision, Writing – review & editing; NA: Software, Writing – review & editing; JJ: Conceptualization, Data curation, Project administration, Writing – review & editing; CHK: Data curation, Software, Writing – review & editing; PZ: Data curation, Software, Writing – review & editing; AG: Conceptualization, Data curation, Writing – review & editing; AK: Conceptualization, Funding acquisition, Project administration, Supervision, Writing – review & editing; MR: Conceptualization, Supervision, Writing – review & editing; JW: Conceptualization, Funding acquisition, Project administration, Supervision, Writing – review & editing; BME: Conceptualization, Funding acquisition, Project administration, Supervision, Writing – review & editing.

## Supplementary Materials

Supplementary Materials
