# Supplementary material for: Contactless Sleep Staging With Radar: A Transfer Learning Approach
Source: IEEE Open J Eng Med Biol. 2026 Feb 23;7:54–62. doi: 10.1109/OJEMB.2026.3667047 (PMC13068128; doi:10.1109/OJEMB.2026.3667047)
Supplement: Supplementary Materials [file supp1-3667047.pdf]

Supplementary Materials

Contactless Sleep Staging with Radar: A Transfer Learning Approach

Daniel Krauss[1*], Robert Richer[1], Nils Albrecht[3], Jelena Jukic[2], Carlos Herrera Krebber[1], Paul Zwiessele[1], Alexander German[2], Alexander Koelpin[3], Martin Regensburger[2], Jürgen Winkler[2], and Bjoern M. Eskofier[1,4]

THESE supplementary materials provide additional analyses and visualizations to complement the findings presented in the main manuscript. Specifically, we provide radar fundamentals that help to understand the measurement principle. Furthermore, we describe the model training procedure in detail including mathematical equation, followed by a subanalysis investigating the classification performance in stable vs. transitional sleep periods. Further comparisons between the radar-only model and the transfer learning approach offer deeper insights into the impact of the model fine-tuning. Additionally, we present a feature correlation matrix comparing the radar-derived features with the concurrently recorded sensor-attached features. Finally, a detailed case study is discussed comparing both models with manually annotated Polysomnography (PSG) labels, illustrating key areas of improvement and the remaining challenges in radar-based sleep staging.

I. Radar Fundamentals

In our study, four 61 GHz Continuous-wave (CW) Doppler radar sensors were placed as an array under the mattress to capture physiological signals relevant for sleep staging (see main manuscript Figure 3). CW Doppler radars operate by transmitting a constant frequency signal to a target and conterminously receiving the reflected signal. Motion of the target relative to the radar causes a frequency shift in the recieved signal due to the Doppler effect [1], [2]. This frequency shift is directly proportional to the velocity of the target along the radar line of sight and allows the radar to capture motion and estimate velocity. Unlike pulse-based radars, CW radars do not provide absolute distance measurements because they lack time-of-flight information. However, they can detect displacements such as physiological movements (e.g., chest wall expansion during breathing or small body movements) by analyzing the phase change of the reflected signal [3].

To fully characterize the phase and amplitude of the received signal, CW radars typically use a quadrature receiver architecture. This involves decomposing the received signal into two components: the in-phase (I) and quadrature (Q) channels. Together, these channels form a complex baseband representation of the received signal, where the phase angle between the I and Q components reflects the displacement of the target over time. This phase information allows for precise tracking of small movements [3]. The higher the operating frequency, the greater the sensitivity to small displacements. At 61 GHz, the radar wavelength is approximately 4.9 mm, making it suitable for capturing subtle motions such as respiration and heart sounds. This wavelength translates to a distance change of 7 µm related to a 1° phase angle rotation. [3].

A. CW baseband signal model (61 GHz)

These The transmitted carrier can be denoted as

$$s_{\text{tx}}(t) = A_{\text{tx}} \cos(\omega_c t + \phi_0^{\text{tx}})$$

with angular frequency $\omega_c = 2\pi f_c$, zero-phase ϕ_0^{tx}), and amplitude A_{tx}.

The backscattered signal s_{rx} with time delay τ and an additional phase offset ϕ_{rx} yields

$$s_{\text{rx}}(t) = A_{\text{rx}} \cos\big(\omega_c(t - \tau + \phi_0^{\text{tx}} + \phi_{\text{rx}}\big).$$

With a receive signal amplitude A_{rx}. Quadrature mixing and low-pass filtering give the complex baseband

$$s_b(t) = I(t) + jQ(t) = A\, e^{j\phi_b(t)}, \qquad \phi_b(t) = \omega_c \tau - \phi_{\text{rx}},$$

with $\tau = \frac{2d}{c}$ and $d = d_0 + x$. For small micromotions $|x| \ll \lambda$,

$$x \approx \frac{\lambda}{4\pi} \Delta\phi_b, \qquad \lambda = \frac{c}{f_c} \ (\approx 4.9 \text{ mm at } 61 \text{ GHz}).$$

The baseband phase $\phi_b(t)$ is proportional to the propagation delay and thus to target distance. Therefore, small displacements x appear as phase modulations and can be retrieved via the linearized relation above [4].

For detecting large body movements, the amplitude variations in the I-channel alone often provide a sufficient proxy for movement intensity. In our analysis, we therefore utilized the I-channel to extract gross body movement features, reducing computational complexity while retaining sensitivity to major motion patterns.

B. Radar Positioning

Our radar setup includes four independent 61 GHz CW radar nodes positioned under the bed, as illustrated in Fig. 2 of the main manuscript. Each node measures reflected signals from a slightly different angle relative to the participant's body position. Due to individual variations in positioning, potential signal attenuation from mattress damping, and subject movement, signal quality can vary across the radar nodes. Previous work from our group has shown that, at 61 GHz, mattress-induced attenuation is moderate (7.1 dB), whereas losses increase substantially at higher carrier frequencies (e.g., 26 dB at 122 GHz), making under-mattress measurements at frequencies substantially above 60 GHz unfeasible [5]. In addition, different mattress types, slatted frame material, and room layout can influence radar signal attenuation and reflection. This can affect signal morphology and generalization to home environments. To increase the robustness of heartbeat detection, we applied a fusion strategy that combines the outputs of all four radar nodes (see main manuscript III.B).

II. Model training

A. Model Input

Each training *sequence* is composed of $S \in \{20, 50, 100\}$ consecutive 30 s epochs (i.e. 10, 25, or 50 min windows, selected by hyper-parameter search). For every epoch we extract the $F = 13$ features listed in Table I in the main manuscript resulting in the input tensor $\mathbf{X} \in \mathbb{R}^{B \times S \times F}$, where B is the batch size. All features were z-normalized prior to model input, ensuring zero mean and unit variance.

To prevent data leakage, sequences were generated separately for each participant, ensuring that data from multiple individuals were not combined within a single sequence.

B. Model Architecture

The model architecture was designed as a single-direction L-layer LSTM with hidden size H that updates its hidden–cell pair $(\mathbf{h}_t, \mathbf{c}_t)$:

$$\begin{bmatrix} \mathbf{i}_t \\ \mathbf{f}_t \\ \mathbf{o}_t \\ \tilde{\mathbf{c}}_t \end{bmatrix} = \sigma\big(\mathbf{W}\,[\mathbf{x}_t, \mathbf{h}_{t-1}]^\top + \mathbf{b}\big), \tag{1}$$

$$\mathbf{c}_t = \mathbf{f}_t \odot \mathbf{c}_{t-1} + \mathbf{i}_t \odot \tilde{\mathbf{c}}_t, \tag{2}$$

$$\mathbf{h}_t = \mathbf{o}_t \odot \tanh(\mathbf{c}_t), \tag{3}$$

where $\sigma(\cdot)$ is the logistic sigmoid and $\odot$ denotes element-wise multiplication. Temporal dynamics are summarised by mean pooling

$$\bar{\mathbf{h}} = \frac{1}{S} \sum_{t=1}^{S} \mathbf{h}_t,$$

followed by a two-layer MLP

$$\mathbf{z} = \mathbf{W}_2\, \phi(\mathbf{W}_1 \bar{\mathbf{h}} + \mathbf{b}_1) + \mathbf{b}_2,$$

and softmax probabilites

$$\mathbf{p} = \mathrm{softmax}(\mathbf{z}),$$

which outputs class probabilities for $C \in \{3, 5\}$ sleep-stage labels.

The number of LSTM layers, sequence length, and dropout rate were treated as hyperparameters, with their specific selection process detailed in Section II-D.

C. Loss function

To address class imbalance (Figure 1), we used a weighted focal loss function [6].

$$\mathcal{L} = -\frac{1}{N} \sum_{n=1}^{N} \alpha_{y_n} \left(1 - p_{y_n}\right)^{\gamma} \log p_{y_n},$$

with focusing parameter $\gamma = 2$ and weights $\alpha_k \propto 1/\mathrm{freq}(k)$. Here, class weights were assigned inversely proportional to class frequency, while the focal loss component further weighted harder-to-classify examples by dynamically down-weighting easy-to-classify instances.

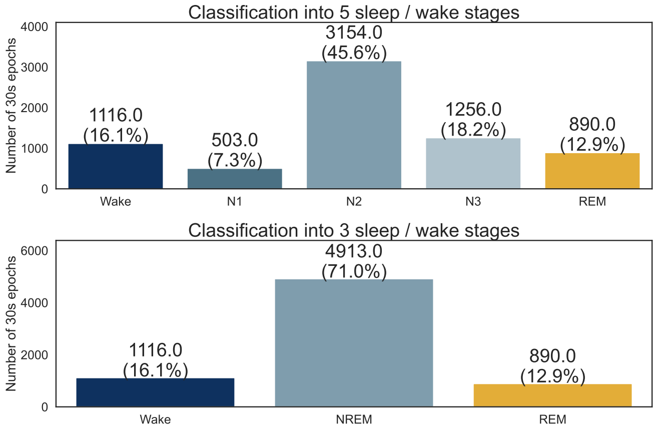

Fig. 1. Sleep stage distribution in the radar dataset, shown for both the five-class scheme according to AASM guidelines (Wake, N1, N2, N3, REM) and the simplified three-class scheme (Wake, NREM, REM).

D. Evaluation

The data was split into 80 % training and 20 % test data on subject level, ensuring that the test set remained completely independent from both the training and validation sets. Within the training set, an additional 20 % was allocated as a validation set for hyperparameter tuning and early stopping.

The Model training was performed for up to 170 epochs using the Adam optimizer [7], an adaptive gradient descent method that dynamically adjusts learning rates (weight-decay 10^{-5}).

We optimized the model performance towards the Matthews Correlation Coefficient (MCC), a robust evaluation metric well-suited for imbalanced classification tasks [8]. The MCC value ranges from -1 (inverse prediction) to 1 (perfect prediction), with 0 indicating random classification performance.

Early stopping was applied that terminated training after five consecutive epochs without validation-loss improvement. The final model was selected as the checkpoint with the lowest validation loss to prevent overfitting. Inputs were z-normalized per batch and gradient norms were clipped to $\|\nabla\|_2 \le 0.5$ to ensure numerical stability.

The hyperparameter optimization was performed using a Tree-structured Parzen Estimator (TPE) algorithm implemented in the Optuna framework [9], exploring a total of 250 trials per model. Table I summarizes the hyperparameter search space and the optimal configurations identified for the radar-only and MESA baseline models, separately for the 3-class and 5-class sleep staging tasks.

III. Cross-modal Feature Correlation Analysis

To quantify the signal-level agreement between radar- and PSG-derived physiological features, we computed per-participant Pearson correlation coefficients between the aligned time series features and pooled them across subjects using a Fisher-z transformation with fixed-effects weighting (n-3). This approach can be used to aggregate correlation coefficients across subjects in multimodal studies [10]. High cross-modal correlation indicates that the radar feature includes similar

Hyperparameter	Search Space	3-Class Classification			5-Class Classification		
		Radar-only	MESA Baseline	Radar-PSG	Radar-only	MESA Baseline	Radar-PSG
Sequence Length	{10, 25, 50} min	50 min	50 min	10 min	10 min	10 min	25 min
Hidden Size	4–700 (step 4)	536	124	380	392	556	256
Number of Layers	1–10	6	3	1	5	6	1
Dropout	0.0–0.5	0.404	0.363	0.112	0.354	0.255	0.093
Learning Rate	1×10^{-6} – 1×10^{-4}	5.93×10^{-6}	3.44×10^{-5}	7.79×10^{-5}	2.85×10^{-5}	6.31×10^{-5}	7.72×10^{-5}

TABLE I. Hyperparameter search space and optimal values identified for the radar-only and MESA baseline models, reported separately for the 3-class and 5-class sleep staging tasks.

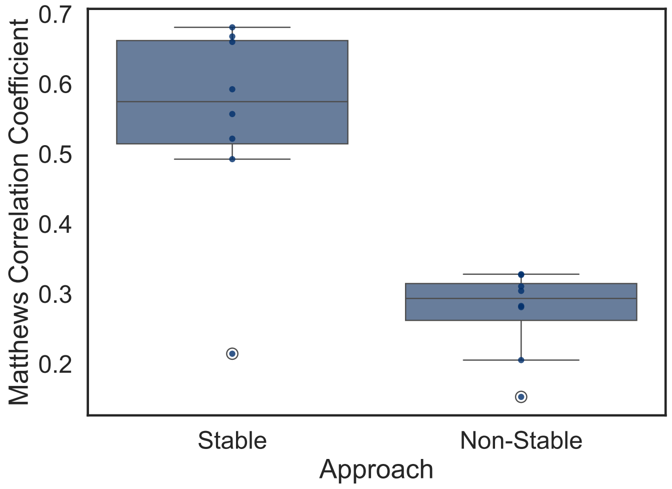

Fig. 2. Comparison of MCC scores for sleep stage classification in stable vs. transitional periods for the transfer learning model.

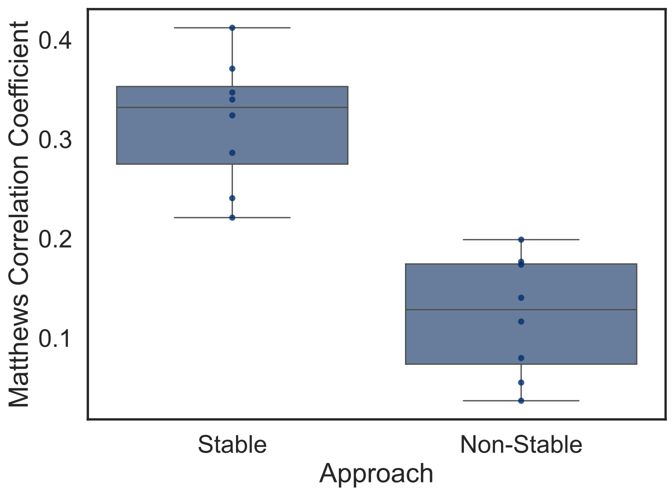

Fig. 3. Comparison of MCC scores for sleep stage classification in stable vs. transitional periods for the radar-only model.

physiological information as its PSG counterpart, while low correlation highlights modality-specific differences (e.g., mechanical heart beat vs. electrical excitation, mattress attenuation, or motion artifacts).

IV. Results & Discussion

A. Analysis of Sleep Stage Transitions

Sleep stage transitions are coined by dynamic physiological changes [11]–[13], making the precise classification of sleep stages more challenging compared to stable sleep periods. To assess model performance in stable and transitional periods, we evaluated the radar-only and transfer learning models separately for stable periods and transitional periods, where transitional periods were defined as occurring within ±2 minutes before or after a change in sleep stage.

The transfer learning model performed much better during stable periods, achieving a median MCC (IQR) of 0.57 (0.15), compared to 0.29 (0.05) during transitions (Figure 2). A similar trend was observed in the radar-only model, with a median MCC (IQR) of 0.33 (0.08) in stable periods and 0.13 (0.10) during transitions (Figure 3).

These findings suggest that classification errors are most prevalent during transitional periods, likely due to the overlap of physiological characteristics from adjacent sleep stages. This observation is consistent with prior work showing greater inter-rater variability even in manual sleep staging during transitions [14], indicating that such changes may not occur as discrete events, but rather reflect gradual physiological shifts.

B. Analysis of Sleep Stage Classification Performance

To further investigate the sleep stage classification performance in both classification schemes, we provide additional box plots.

In sleep staging according to AASM standards (Wake, N1, N2, N3, REM)) we observed a strong increase across all performance metrics when comparing the both the radar-only and the psg-only models to the respective transfer learning models (Figure 4). In the second experiment (Wake, NREM, REM) the transfer learning models showed a considerable, though not as drastically improvement over the radar and psg-only approaches as observed within the first experiment. This indicates that the primary strength of the transfer learning approach is its ability to further differentiate the substage of NREM (N1, N2, N3), rather than in broader stage separation (Figure 5).

C. Correlations between PSG-derived and radar-derived features

Participant-wise Fisher-z-pooled correlations showed the strongest agreement for median NN intervals (150-s window

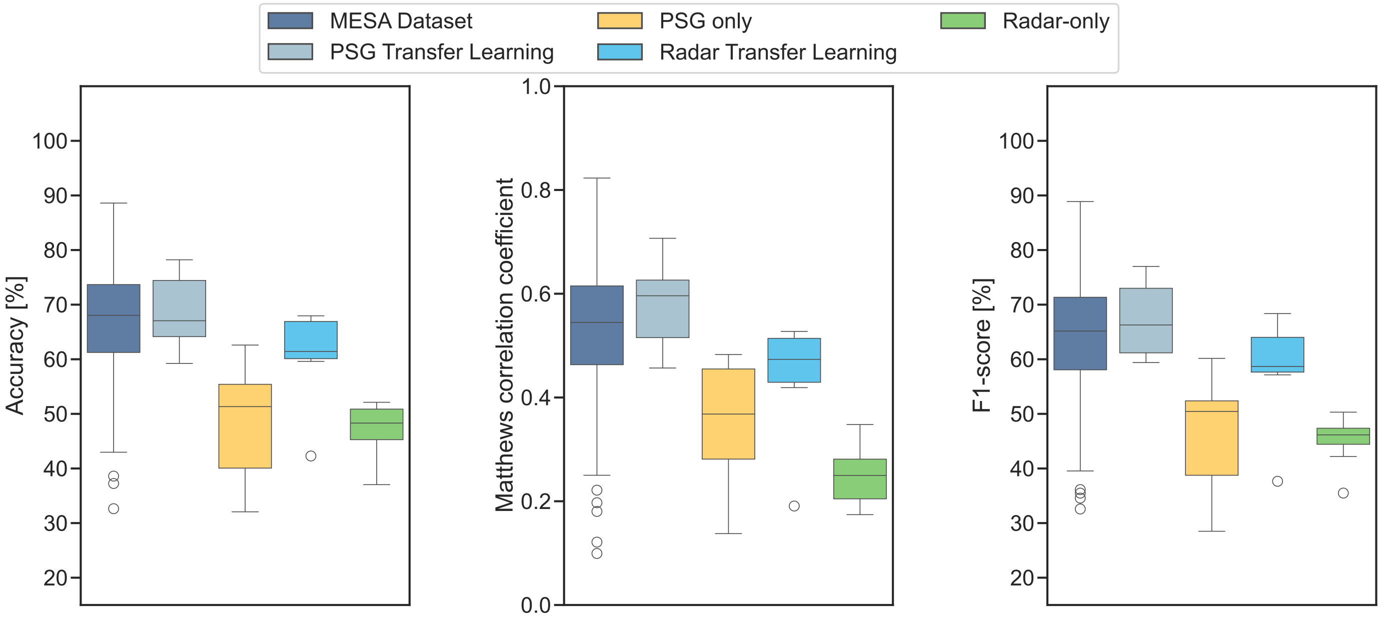

Fig. 4. Performance of sleep-stage classification according to AASM standards (Wake / N1 / N2/ N3 / REM) for the MESA model, the radar-only model and the transfer learning approach.

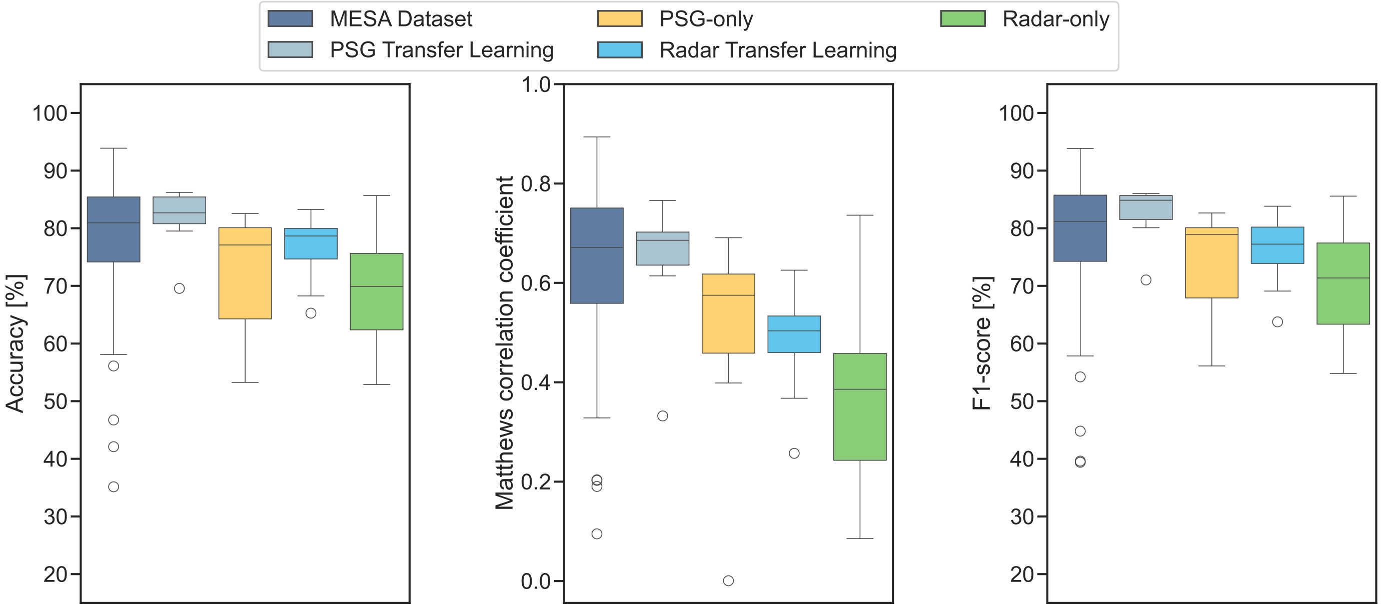

Fig. 5. Performance of Wake / NREM / REM classification for the MESA model, the radar-only model and the transfer learning approach.

$r = 0.80$, 30-s window: $r = 0.65$). Respiratory variability also showed moderate to strong agreement (e.g., MedianBB $r = 0.64$) and movement correlated well ($r = 0.61$).

In contrast, HRV spectral indices (LF, HF, LF/HF, total power) exhibited weak cross-modal correlations ($r = 0.01$ – 0.20). This weaker correlation is probably due to the cardio-mechanical measurement, as well as motion-artifacts and mattress attenuation. Scatter plots for median NN, MedianBB, and movement are shown in the main manuscript (Figure 5), the full Fisher-z–pooled matrix is provided in Figure 7.

D. Case Study to Analyze Radar-Only and Transfer Learning Approach by comparing the Hypnograms

To qualitatively assess model performance, we compared the sleep stage predictions of the radar-only and transfer learning models against the gold-standard PSG labels. Figure 6 presents a hypnogram of a representative participant (ID 40), highlighting regions where the transfer learning model (dashed red line) and radar-only model (dotted gray line) deviate from the ground truth (solid blue line).

The transfer learning model demonstrated a closer alignment with PSG annotations, particularly across sustained periods of N2 and N3 sleep. In contrast, the radar-only model exhibited

more frequent misclassifications, notably confusing N2 with Wake and REM with N2, especially during transitional phases. Particularly during stable N3 sleep phases (e.g., between 00:30 – 01:30 and 02:30 – 03:30), the transfer learning model captured the participant's deep sleep more consistently, likely due to its superior ability to capture long-term stability in sleep stages. However, the transfer learning model introduces occasional errors compared to the radar-only model, especially when underestimating N2 sleep directly before or after sleep stage transitions.

References

[1] D. W. Fife, "Image Frequency Rejection in a Radio Direction Finder," *IEEE Transactions on Aerospace and Navigational Electronics*, vol. ANE-10, no. 2, pp. 128–132, Jun. 1963. [Online]. Available: https://ieeexplore.ieee.org/document/4502099

[2] W. D. Boyer, "A Diplex, Doppler Phase Comparison Radar," *IEEE Transactions on Aerospace and Navigational Electronics*, vol. ANE-10, no. 1, pp. 27–33, Mar. 1963. [Online]. Available: https://ieeexplore.ieee.org/document/4502075

[3] D. Krauss, L. Engel, T. Ott, J. Bräunig, R. Richer, M. Gambietz, N. Albrecht, E. M. Hille, I. Ullmann, M. Braun, P. Dabrock, A. Kölpin, A. D. Koelewijn, B. M. Eskofier, and M. Vossiek, "A Review and Tutorial on Machine Learning-Enabled Radar-Based Biomedical Monitoring," *IEEE Open Journal of Engineering in Medicine and Biology*, vol. 5, pp. 680–699, 2024, conference Name: IEEE Open Journal of Engineering in Medicine and Biology. [Online]. Available: https://ieeexplore.ieee.org/document/10520876#citations

[4] N. C. Albrecht, D. Langer, D. Krauss, R. Richer, L. Abel, B. M. Eskofier, N. Rohleder, and A. Koelpin, "EmRad: Ubiquitous Vital Sign Sensing Using Compact Continuous-Wave Radars," *IEEE Open Journal of Engineering in Medicine and Biology*, vol. 5, pp. 725–734, 2024, conference Name: IEEE Open Journal of Engineering in Medicine and Biology. [Online]. Available: https://ieeexplore.ieee.org/document/10577086

[5] N. C. Albrecht, J. P. Weiland, D. Langer, M. Wenzel, and A. Koelpin, "Characterization of the Influence of Clothing and Other Materials on Human Vital Sign Sensing using mmWave Radar," in *2023 53rd European Microwave Conference (EuMC)*, Sep. 2023, pp. 428–431. [Online]. Available: https://ieeexplore.ieee.org/document/10290459

[6] T.-Y. Lin, P. Goyal, R. Girshick, K. He, and P. Dollar, "Focal Loss for Dense Object Detection," 2017, pp. 2980–2988. [Online]. Available: https://openaccess.thecvf.com/content_iccv_2017/html/Lin_Focal_Loss_for_ICCV_2017_paper.html

[7] D. P. Kingma and J. Ba, "Adam: A Method for Stochastic Optimization," *arXiv:1412.6980 [cs]*, Jan. 2017. [Online]. Available: http://arxiv.org/abs/1412.6980

[8] D. Chicco and G. Jurman, "The advantages of the Matthews correlation coefficient (MCC) over F1 score and accuracy in binary classification evaluation," *BMC Genomics*, vol. 21, no. 1, p. 6, Jan. 2020. [Online]. Available: https://doi.org/10.1186/s12864-019-6413-7

[9] T. Akiba, S. Sano, T. Yanase, T. Ohta, and M. Koyama, "Optuna: A Next-generation Hyperparameter Optimization Framework," in *Proceedings of the 25th ACM SIGKDD International Conference on Knowledge Discovery & Data Mining*, ser. KDD '19. New York, NY, USA: Association for Computing Machinery, Jul. 2019, pp. 2623–2631. [Online]. Available: https://dl.acm.org/doi/10.1145/3292500.3330701

[10] D. M. Corey, W. P. Dunlap, and M. J. Burke, "Averaging Correlations: Expected Values and Bias in Combined Pearson rs and Fisher's z Transformations," *The Journal of General Psychology*, vol. 125, no. 3, pp. 245–261, Jul. 1998, publisher: Routledge _eprint: https://doi.org/10.1080/00221309809595548. [Online]. Available: https://doi.org/10.1080/00221309809595548

[11] T. Porkka-Heiskanen, K.-M. Zitting, and H.-K. Wigren, "Sleep, its regulation and possible mechanisms of sleep disturbances," *Acta Physiologica*, vol. 208, no. 4, pp. 311–328, 2013, _eprint: https://onlinelibrary.wiley.com/doi/pdf/10.1111/apha.12134. [Online]. Available: https://onlinelibrary.wiley.com/doi/abs/10.1111/apha.12134

[12] A. N. Goldstein and M. P. Walker, "The role of sleep in emotional brain function," *Annu Rev Clin Psychol*, vol. 10, pp. 679–708, 2014.

[13] D. Moser, P. Anderer, G. Gruber, S. Parapatics, E. Loretz, M. Boeck, G. Kloesch, E. Heller, A. Schmidt, H. Danker-Hopfe, B. Saletu, J. Zeitlhofer, and G. Dorffner, "Sleep Classification According to AASM and Rechtschaffen & Kales: Effects on Sleep Scoring Parameters," *Sleep*, vol. 32, no. 2, pp. 139–149, Feb. 2009, tex.ids= Moser2009a. [Online]. Available: https://doi.org/10.1093/sleep/32.2.139

[14] Y. J. Lee, J. Y. Lee, J. H. Cho, and J. H. Choi, "Interrater reliability of sleep stage scoring: a meta-analysis," *Journal of Clinical Sleep Medicine*, vol. 18, no. 1, pp. 193–202, publisher: American Academy of Sleep Medicine. [Online]. Available: https://jcsm.aasm.org/doi/full/10.5664/jcsm.9538

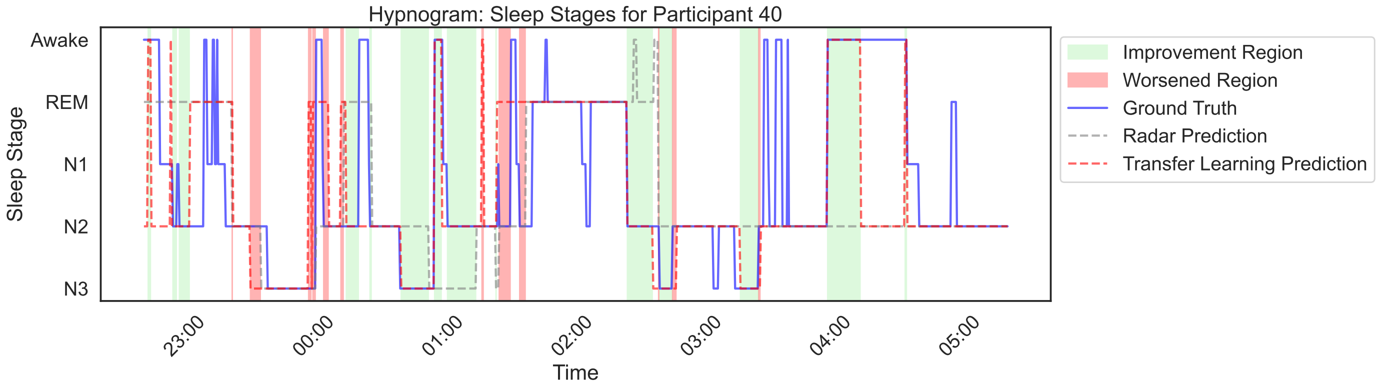

Fig. 6. Comparison of sleep stage predictions from the radar-only model and transfer learning model against PSG ground truth for a representative participant (ID 40). Green shading indicates improved classification by transfer learning, while red shading highlights worsened predictions.

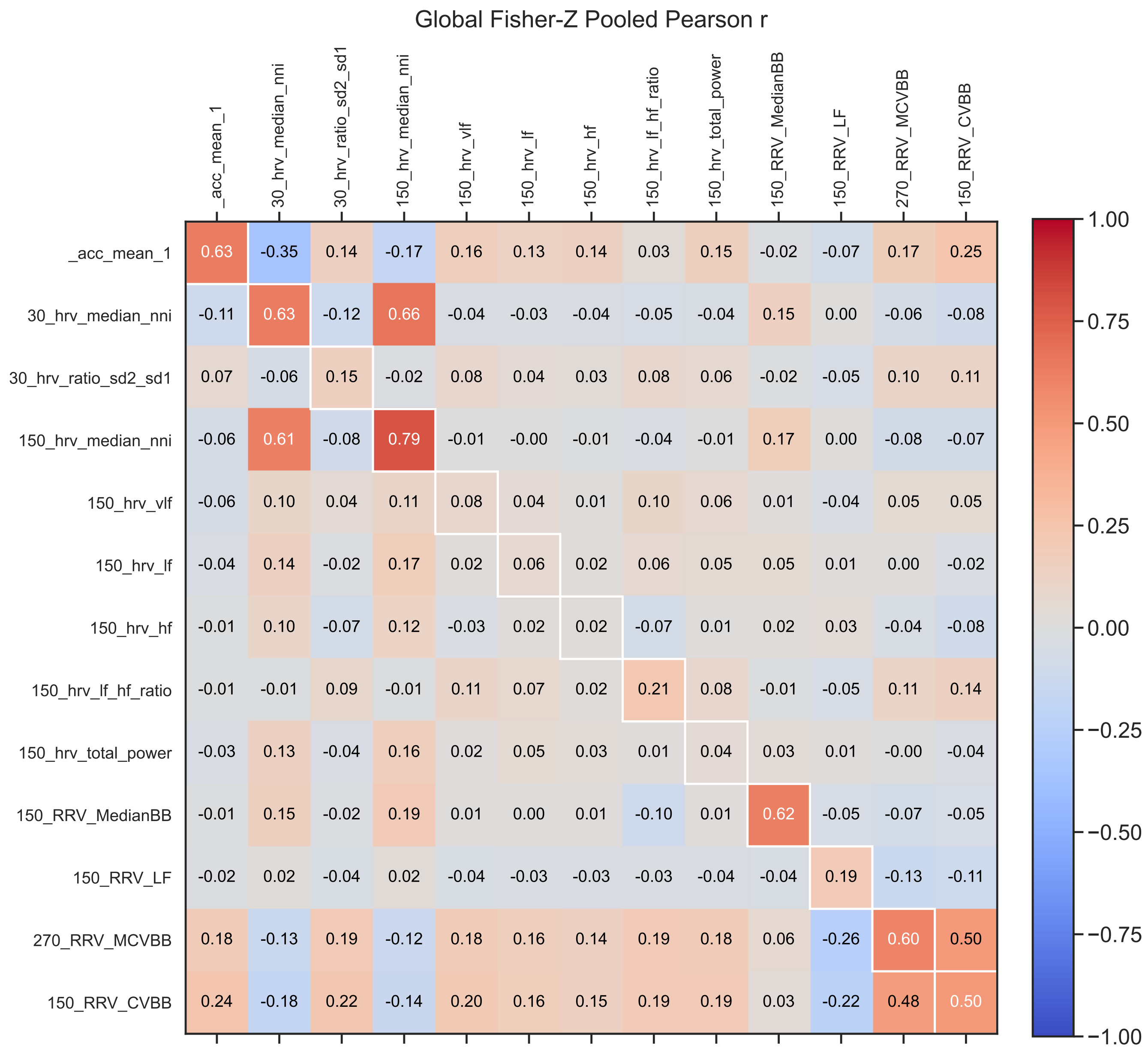

Fig. 7. Global Fisher-z pooled Pearson correlations between radar- and PSG-derived features on aligned 30-s epochs. rows = radar, columns = PSG.
